# Characterizing early embryonic development of Brown Tsaiya Ducks (*Anas platyrhynchos*) in comparison with Taiwan Country Chicken (*Gallus gallus domestics*)

**DOI:** 10.1371/journal.pone.0196973

**Published:** 2018-05-09

**Authors:** Chompunut Lumsangkul, Yang-Kwang Fan, Shen-Chang Chang, Jyh-Cherng Ju, Hsin-I. Chiang

**Affiliations:** 1 Department of Animal Science, National Chung Hsing University, Taichung, Taiwan, ROC; 2 Kaohsiung Animal Propagation Station, Livestock Research Institute, Council of Agriculture, Pingtung, Taiwan, ROC; 3 Graduate Institute of Biomedical Sciences, China Medical University, Taichung, Taiwan, ROC; 4 Core Lab for Stem Cell Research, Medical Research Department, China Medical University Hospital, Taichung, Taiwan, ROC; 5 Department of Bioinformatics and Medical Engineering, Asia University, Taichung, Taiwan, ROC; Gaziosmanpasa University, TURKEY

## Abstract

Avian embryos are among the most convenient and the primary representatives for the study of classical embryology. It is well-known that the hatching time of duck embryos is approximately one week longer than that of chicken embryos. However, the key features associated with the slower embryonic development in ducks have not been adequately described. This study aimed to characterize the pattern and the speed of early embryogenesis in Brown Tsaiya Ducks (BTD) compared with those in Taiwan Country Chicken (TCC) by using growth parameters including embryonic crown-tail length (ECTL), primitive streak formation, somitogenesis, and other development-related parameters, during the first 72 h of incubation. Three hundred and sixty eggs from BTD and TCC, respectively, were incubated at 37.2°C, and were then dissected hourly to evaluate their developmental stages. We found that morphological changes of TCC embryos shared a major similarity with that of the Hamburger and Hamilton staging system during early chick embryogenesis. The initial primitive streak in TCC emerged between 6 and 7 h post-incubation, but its emergence was delayed until 10 to 13 h post-incubation in BTD. Similarly, the limb primordia (wing and limb buds) were observed at 51 h post-incubation in TCC embryos compared to 64 h post-incubation in BTD embryos. The allantois first appeared around 65 to 68 h in TCC embryos, but it was not observed in BTD embryos. At the 72 h post-incubation, 40 somites were clearly formed in TCC embryos while only 32 somites in BTD embryos. Overall, the BTD embryos developed approximately 16 h slower than the chicken embryo during the first 72 h of development. To our best knowledge, this is the first study to describe two distinct developmental time courses between TCC and BTD, which would facilitate future embryogenesis-related studies of the two important avian species in Taiwan.

## Introduction

Advantages of using avian embryos for the study of embryology lie in its ease of availability, manipulation and observation, as well as the relatively low cost of the eggs. It has been popular to use avian eggs for the study of embryogenesis including organogenesis and morphogenesis, and hence the developmental stages are established in several avian species, such as chickens [1], quails [2], emus [3] and geese [4]. Although these species share some major features of early embryogenesis, they certainly possess species-specific characteristics of embryonic stages during the course of development. Hamburger and Hamilton [1] have described a series of characterized embryonic stages in chicken, in which a clearly defined 46-stage series is established. Ainsworth *et al*. [2] also described a whole set of definitive developmental stages in Japanese quail embryos, in which little differences can be tell from chicken embryogenesis during the early development. In contrast, Nagai *et al*. [3] have reported that emu embryos require approximately a 2- to 3-time longer incubation to reach the equivalent stages of chicken embryos.

It is generally accepted that differences in developmental time course, such as hatching time, hatched size and chick maturity among avian species are attributed to various genetics and/or environmental factors during development [5, 6]. Recently, studies on early embryonic stages have been reported in the Pekin duck [7–10] and mallard [11], but the precise timeline and the stages of embryonic development relative to chicken embryos have not been clearly described. Although duck embryos are known to take longer incubation time to reach their full-term development than do chicken embryos [12], the detailed embryonic features have not been characterized side-by-side. To advance our understanding in avian embryogenesis, therefore, it is necessary to closely examine and determine the precise timing of the developmental events between the two species.

In the present study, we aimed to characterize and standardize the pattern of early embryogenesis in the Brown Tsaiya Duck (BTD) in comparison with that of the Taiwan Country Chicken (TCC) and the HH staging system by using several representative growth parameters, such as primitive streak formation, notochord development, embryonic crown-tail length (ECTL), somite numbers, and other development-associated phenomena during the first 72 h of development.

## Materials and methods

### Eggs used and conditions of incubation

A total of 720 fertilized eggs were used in this study, in which 360 eggs were collected from BTDs and another 360 eggs were from TCCs (NCHU B line match × NCHU S line). To accumulate enough number of fertilized eggs, all these eggs were collected and stored at 15 to 18°C (with 65% relative humidity) less than 3 days before use, based on the recommended storage condition by previous studies [13–16]. During this short period of storage, these eggs ceased embryonic development. To start development, adequate numbers of eggs were transferred from storage condition to an incubator set at 37.2°C. The whole study was carried out in strict accordance with the guideline recommended and approved by the Institutional Animal Care and Use Committee (IACUC) of the National Chung Hsing University (Permit number: 100–02).

### Preparation of embryos

The blastoderm of incubated embryos was isolated according to the method described by Chapman *et al*. [17]. Briefly, the eggshell was opened to separate the yolk from the albumen. The thick albumen covering the embryo was removed using a piece of folded filter paper. A filter paper ring with an inner diameter of one cm and an outer diameter of three cm was placed onto the yolk to set the embryo at the center hole of the filter paper, and to absorb liquid from the surface of the yolk. The perivitelline layer was then cut along the perimeter of the outer edge of the filter paper. The filter paper with the embryo was then carefully pulled off by a pair of forceps, and transferred to a petri dish containing saline to separate the germ layer from the yolk. All yolk remnants were gently washed off with saline, and the attached embryos with filter paper were finally placed on a glass slide for assessing embryonic stages under a trinocular stereomicroscope (SMZ-2T, Nikon, Japan).

To observe the emergence of somites, embryos were stained with a minimal amount of 0.5% neutral red (Sigma-Aldrich, USA; N-4638) in Hank’s balanced salt solution (HBSS) to facilitate subsequent visualization.

### Staging and sampling of embryos

The age of embryos was defined chronologically from the onset of incubation up to the end point of observation. Due to the first 72 h of embryogenesis being a critical stage to observe early embryo differentiation in avian species, in the present study, embryos were first analyzed or staged hourly up to 72 h post-incubation within 1 h after removing from the incubator. Three fertilized eggs of each species, i.e., TCC and BTD, were used for the assessment on each time point during the first 24 h of incubation. For the determination of somite numbers and other embryonic traits, six fertilized eggs were used for each time point after the 24 h of incubation due to the increased growth rate and variations. For embryonic development, the Hamburger and Hamilton (HH) staging system was used as a parallel comparison with BTD and TCC embryos.

Embryo stages were defined and distinguished based on the emergence of embryonic structures including headfold, neural fold, primary optic vesicles, paired heart primordia, the nervous system (three primary brains and five neuromeres), the limb primordia (wing and leg buds) and the allantois.

### Measurement of growth parameters

Various growth parameters including the primitive streak, the notochord, numbers of somites, and the ECTL were recorded hourly during the first 72 h of development. The ECTL was measured from top of the crown to the tail-bud along the dorsal midline of the embryo. The length of primitive streak was measured from the sickle-shaped region at the interface between the area opaca and area pellucida; such structure can extend to the posterior midline region of the embryo. The length of the notochord was measured from the anterior of Hensen's node to the prosencephalon. The numbers of somite pairs located in the middle of embryonic axis were counted hourly during the entire period of incubation.

The whole mount embryos were photographed using an eyepiece C-mount camera (5 MP linux, Tucsen, China) attached to a trinocular stereomicroscope (SMZ-2T, Nikon, Japan) and processed by the software package Tsview 7 (Bioimager, Canada). Micrographs of embryos were prepared and repositioned with enhanced lighting by using a graphic editing program (Adobe Photoshop, USA).

### Statistical analyses

Comparisons of growth parameters between TCC and BTD embryos were made by using analysis of variance with ANOVA procedure of SAS Enterprise Guide Software V. 9.4 (SAS Institute, Cary, NC, USA). Least square means were compared by using Tukey’s test. Orthogonal polynomial contrast (linear and quadratic) was used to detect the hourly effect of incubation on variables by t-test with IBM SPSS Statistics V. 20 software for windows (SPSS Inc., Chicago). A probability level of *P* < 0.05 was considered as statistically significant.

## Results

### Gross parameters of BTDs and TCCs at the 72 h post-incubation

In general, the average size of the BTD eggs was proportionally larger than that of the TCC eggs. The average weights of BTD and TCC eggs used in this study were 66.1 ± 4.2 g and 52.9 ± 3.8 g, respectively. The fertilization rates at 72 h of incubation were 90.3% in TCCs and 85.7% in BTDs, with 2% abnormal embryos in TCC embryos and 0.67% in BTD embryos. The average hatching days of BTD and TCC embryos were 28 and 21 days, respectively, post-incubation.

### Embryonic features related to gastrulation of BTD and TCC embryos

**Fig 1:**
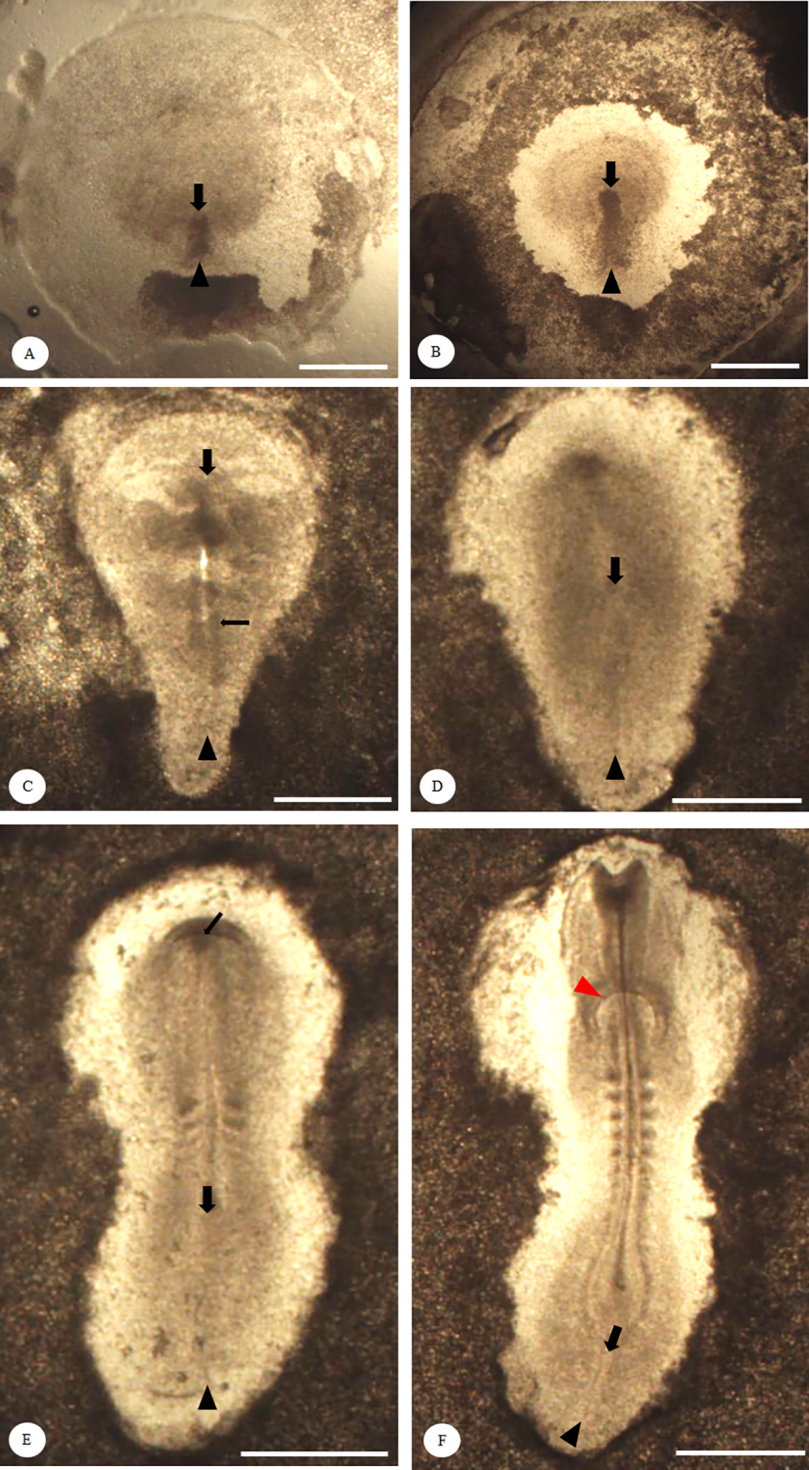
Development and regression of the primitive streak in Brown Tsaiya Duck during the first 72 h of incubation. Representative figures show the primitive streak of Brown Tsaiya Duck (BTD) embryos. Black arrowheads indicate the beginning of the primitive steak and black arrows indicate the growing or regressing end of the streak. (A) The initial streak can be observed by 10–13 h post-incubation or earlier; (B) the initial primitive streak elongates into the intermediate streak by 19–24 h post-incubation; (C) a full-length streak can be observed, with a clearly formed neural groove (thin black arrow) by 25–27 h post-incubation; (D-F) after reaching its full length (D), the streak starts to regress by 28–42 h post-incubation, along with the formation of 3–4 somite pairs, pharyngeal endoderm (thin black arrow) (E) and heart primordia (red arrowhead) with more than 6 somite pairs (F). Bright field, scale bar = 0.5 mm.

Embryonic development in BTDs and TCCs were observed hourly during the incubation in comparison with the defined developmental phases of the HH staging system (Table 1). During gastrulation, we found that formation of the primitive streak in TCC embryos was approximately one stage earlier than that of BTD embryos. The primitive streak in both TCC and HH embryos first appeared at 6–7 h post-incubation, but it was still not discernible until 10–13 h in BTD embryos. The length of primitive streak was fully extended around 18 h post-incubation in TCC and HH embryos, but the time for reaching its full length was delayed until 25 h post-incubation in BTD embryos. Regression of the primitive streak in TCCs and BTDs started at 23 h and 28 h post-incubation, respectively. Representative embryos at various phases of primitive streak formation are shown in Fig 1. The neural fold and the first pair of somites of chick embryos normally occurred during 23–26 h (HH system) and 24–26 h (TCC embryos) post-incubation, which were not formed until 32–34 h post-incubation in BTD embryos.

**Table 1:** The emerging time of embryonic structures in Taiwan Country Chicken (TCC) and Brown Tsaiya Ducks (BTD) during the first 72 h of incubation in comparison with the HH* staging system.

	Hour post-incubation		
Embryonic structures	HH*	TCC	BTD
Two-layered blastoderm (HH1; D13) **	0–5	0–5	1–12
Primitive streak (HH2; D14)	6–7	6–7	10–13
Intermediate primitive streak (HH3; D15)	12–13	12–13	19–24
Full-length primitive streak (HH4; D16)	18–19	18–19	25–27
Regressing primitive streak	N/A	23–39	28–42
Headfold formation (HH6; D19)	23–25	22–25	31–36
First somite pair (HH7; D20)	23–26	24–26	32–34
Neural fold (HH7; D21)	23–26	24–26	32–34
Primary optic vesicles (HH9; D22)	29–33	27–32	36–42
Paired heart primordia (HH9; D22)	29–33	26–32	36–42
Nervous system			
Three primary brains (HH10; D23)	33–38	33–39	43–46
Five neuromeres (HH11; D24)	40–45	40–45	46–52
Limb primordia; wing and leg buds (HH17; D30)	51–64	51–64	64–72
Allantois (HH19; D32)	65–72	68–72	N/A

*Hamburger-Hamilton staging system

** The embryonic structures refer to the stages from Hamburger and Hamilton (1915) and Dupuy *et al*. (2002), designated as D-staging or D.

N/A: data not available.

**Fig 2:**
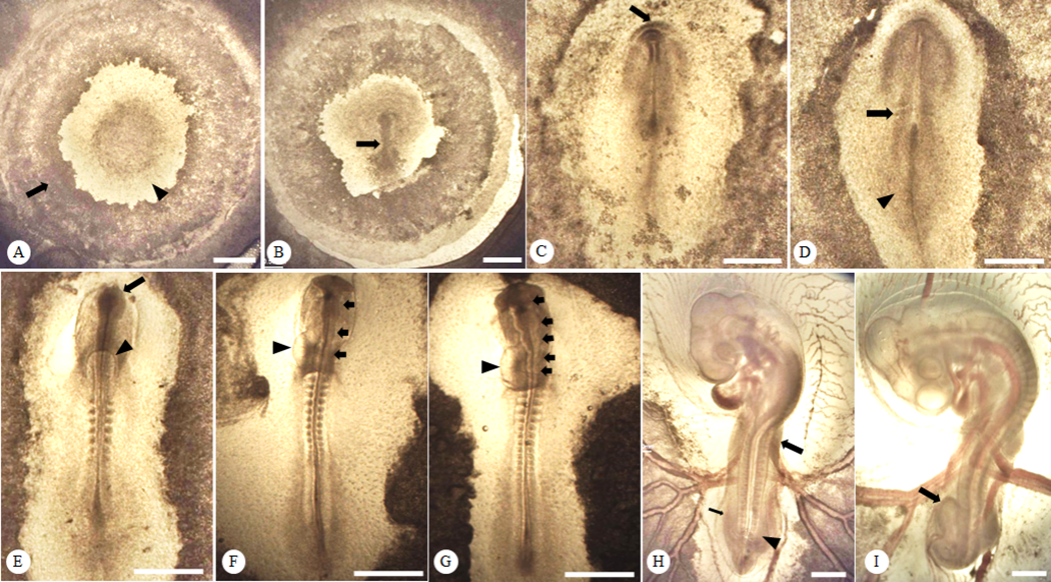
Developmental structures and rudiments of embryonic features during the first 72 h of incubation. Representative figures show the structures of Taiwan Country Chicken (TCC) embryos. (A) The area opaca (arrow) and the area pellucida (arrowhead) are distinct at 5 h post-incubation; (B) the primitive streak (arrow) appears at 12 h in TCC post-incubation; (C) the headfold (arrow) becomes visible at 25 h post-incubation; (D) the initial pair of somites (arrow) and neural plate (arrowhead) first appear at 26 h post-incubation; (E) the primary optic vesicles (arrow) and the paired heart primordia (arrowhead) start to form at 30 h post-incubation; (F) the three primary brain rudiments (arrows) are visible at 33 h post-incubation along with the developing heart primordia (arrowhead) at the 13-somite stage; (G) the five neuromeres (arrows) are distinguishable at 42 h post-incubation with a relatively developed heart (arrowhead); (H) the wing (arrow) and leg buds (arrowhead) develop at 57 h post-incubation and the allantois is barely visible (thin arrow); (I) a prominently enlarged allantois (arrow) can be identified at 72 h post-incubation. Bright field, scale bar = 0.5 mm.

At the early phase of organogenesis, the first indication of heart formation was defined by a paired primordia along with the primary optic vesicles, which arose at 36 h post-incubation in BTD embryos, but it was 9–11 h earlier in chicken embryos of TCC (26–32 h) and the HH staging system (29–33 h, Table 1). In the development of the nervous system, the appearance of three primary brain vesicles occurred around the same time (33–39 h) in both embryos of TCC and the HH system, but its occurrence delayed by 10 h in BTD embryos (43–46 h, Table 1). A similar delay was also observed in the sprouting of limb primordia (wing and leg buds), where the emergence was observed at 51 h post-incubation in TCC embryos compared to 64 h post-incubation in BTD embryos. On the third day of incubation (65–68 h), the allantois was first emerged in chicken embryos but not in BTD embryos. A summary of major embryonic features at various developing stages is shown in Fig 2.

### Growth of BTD and TCC embryos

The length of primary primitive streak, intermediate (elongating) streak, the full length at 18–19 h and the regressed length at 28–42 h were significantly different (*P* < 0.05) between TCC and BTD embryos (Table 2). Except for its full length at 25–27 h and regressed length at 23–39 h, significant differences were observed in the primitive streak measurements between these two species. For somitogenesis, the TCC embryo formed *circa* 40 pairs of somites but only 32 pairs were observed in BTD embryos (*P* < 0.0001) after 72 h post-incubation.

**Table 2:** Comparison of major developmental parameters between Taiwan Country Chicken (TCC) and Brown Tsaiya Duck (BTD) embryos during the first 72 h post-incubation.

	Species		
Morphological measurements or embryonic structures by hours of incubation	TCC	BTD	*P* value
Crown-to-tail length (ECTL) at first 72 h, mm	8.93^a^	6.23^b^	0.0014
Primitive streak length*			
6–7 h post-incubation^1^	1.17^a^	0.00^b^	<0.0001
10–13 h post-incubation^2^	1.56^a^	0.68^b^	<0.0001
Intermediate primitive streak length*			
12–13 h post-incubation^1^	1.72^a^	0.76^b^	0.0009
19–24 h post-incubation^2^	2.64^a^	1.07^b^	<0.0001
Full-length primitive streak*			
18–19 h post-incubation^1^	2.55^a^	0.92^b^	<0.0001
25–27 h post-incubation^2^	1.66	1.46	0.2874
Regressing primitive streak*			
23–39 h post-incubation^1^	1.15	0.99	0.1834
28–42 h post-incubation^2^	1.00^a^	0.55^b^	<0.0001
No. of somites formed during the first 72 h	40^a^	32^b^	0.0001

* Values of the measurements are adjusted proportionally by the egg weight of embryos.

^1^ Hours of incubation are based on the emerging time of embryonic structures in TCC embryos.

^2^ Hours of incubation are based on the emerging time of embryonic structures in BTD embryos.

^a, b^ Within the row, means without the same superscript differed (*P* < 0.05).

**Fig 3:**
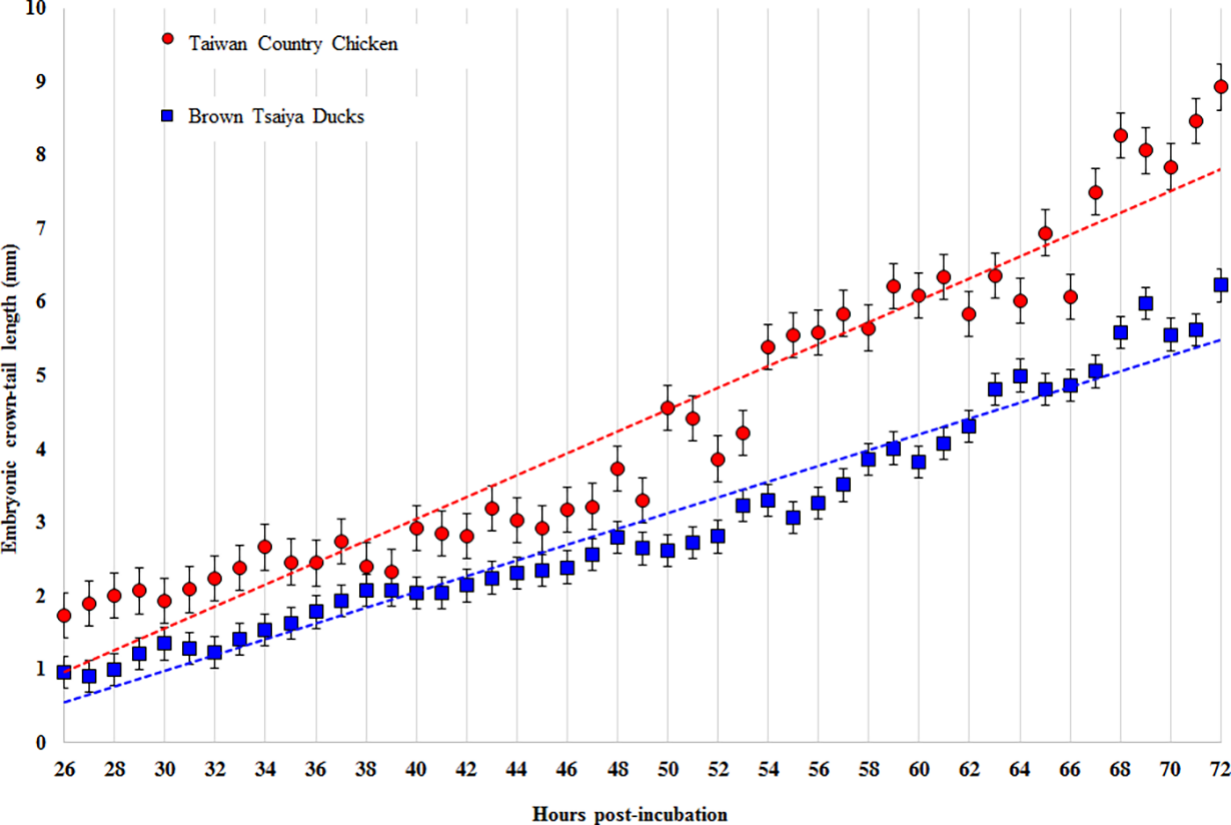
Establishment of linear regression lines between incubation time and embryonic crown-tail length (ECTL). The rates of embryo growth in Brown Tsaiya Ducks (blue dot line; y = 0.107x - 2.2185, R^2^ = 0.9522) and Taiwan Country Chicken (red dot line; y = 0.1486x - 2.8836, R^2^ = 0.9269) can be accurately predicted by each own regression line. Values are presented as mean ± SD in both species.

During the first 72 h post-incubation, there was a significant difference in the ECTL between TCC and BTD embryos (*P* = 0.0014). As shown in Fig 3, the ECTL ranged from 1.74 to 8.93 mm in TCC embryos and from 0.91 to 6.23 mm in BTD embryos during the first 72 h post-incubation. The increase of ECTL showed a strong positive correlation with the time of incubation in both TCC (R^2^ = 0.81) and BTD (R^2^ = 0.79) embryos (*P* < 0.0001). The BTD embryos showed a slower growth of ECTL than that of TCC as determined by a lower slope (R^2^ = 0.93 *vs*. 0.95, *P* < 0.05) in regression lines.

**Fig 4:**
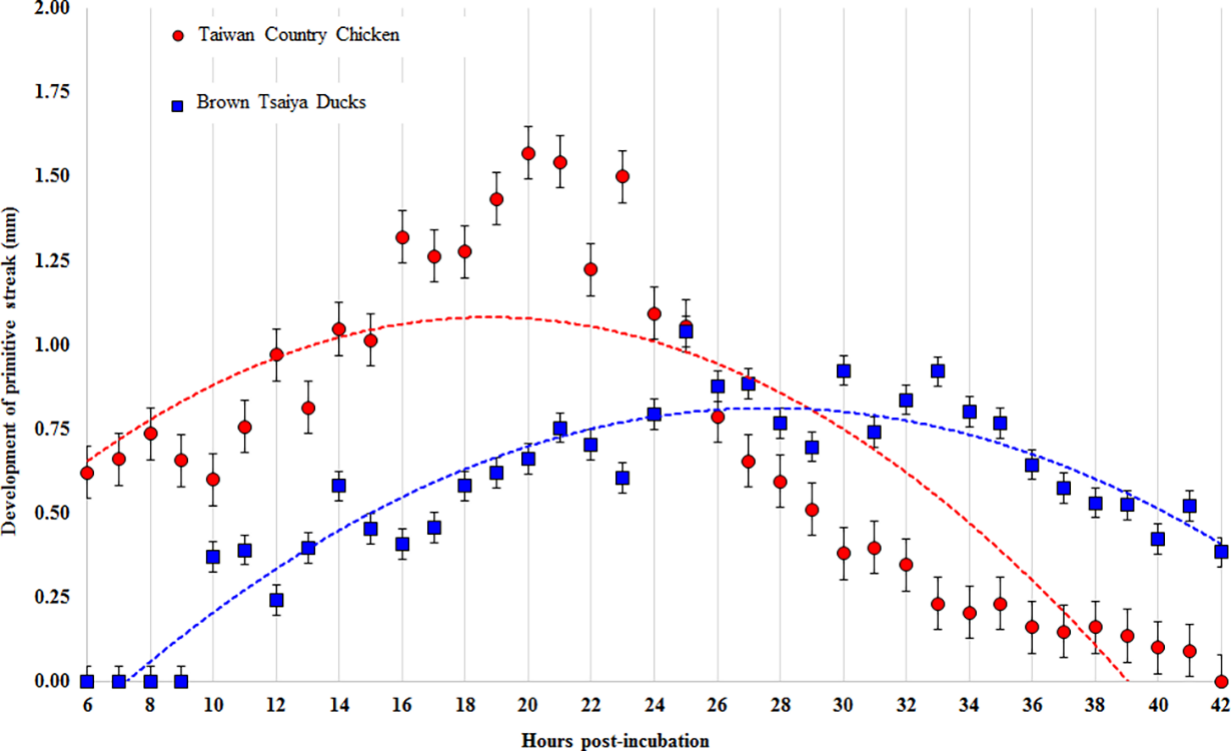
Quadratic regression lines between incubation time and development of the primitive streak in Taiwan Country Chicken (TCC) and Brown Tsaiya Ducks (BTD). The developing primitive streak of embryos in BTD (blue dot line; y = -0.002x^2^ + 0.1079x - 0.6786 R^2^ = 0.8716) and TCC (red dot line; y = -0.0026x^2^ + 0.0985x + 0.1585 R^2^ = 0.7266) can be well-represented with each quadratic regression line. Values are presented as mean ± SD in both species.

Fig 4 shows quadratic relationship between the development of primitive streak and time of incubation in BTD and TCC embryos. Increasing lengths of the primitive streak of TCC *vs*. BTD embryos were observed initially from 0.64 *vs*. 0.44 mm to its full length over 1.36 *vs*. 0.93 mm, and the primitive streak then became shortest at 0.66 *vs*. 0.67 mm by the end of the regression phase, respectively. Development and regression rates of the primitive streak at any given time points were generally slower in BTD embryos than those in TCC embryos as indicated by tangents of the two quadratic regression lines (R^2^: 0.87 *vs*.0.73).

**Fig 5:**
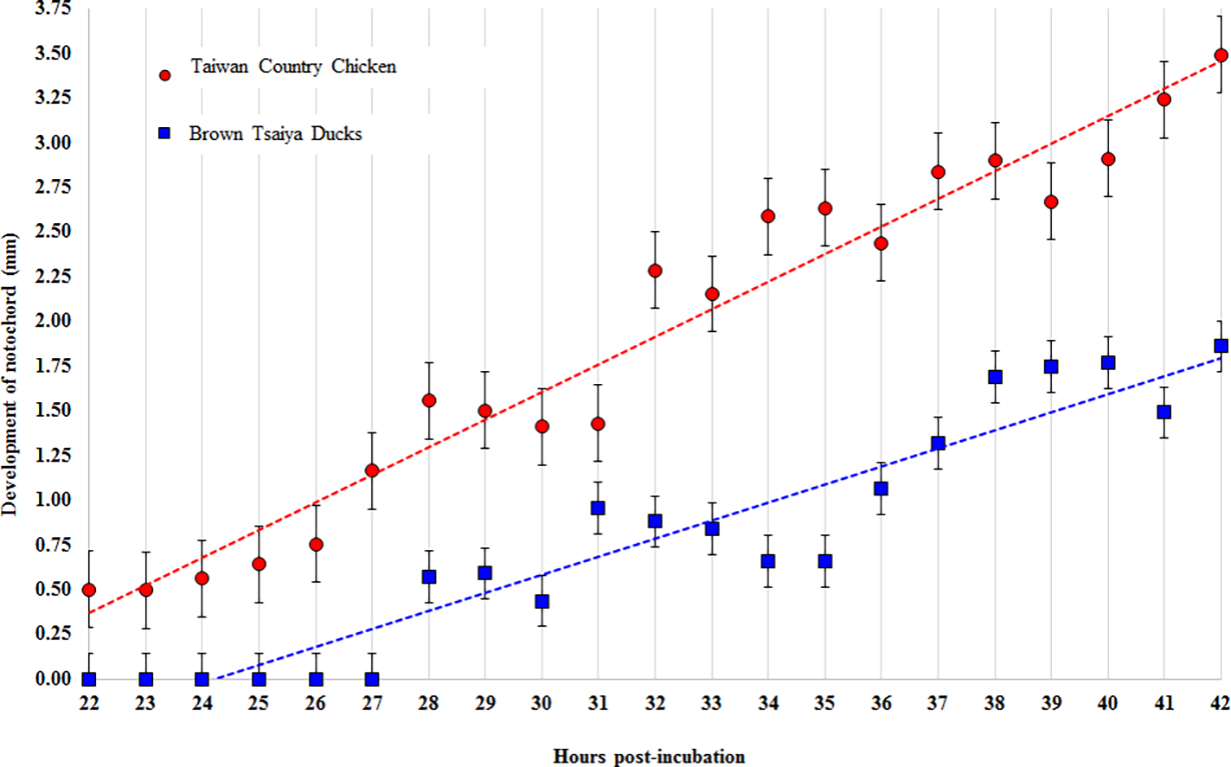
Linear regression lines between incubation time and development of the notochord in Taiwan Country Chicken (TCC) and Brown Tsaiya Duck (BTD). Values are presented as mean ± SD in Brown Tsaiya Ducks (blue dot line; y = 0.1008x - 2.4389 R^2^ = 0.8994) and Taiwan Country Chicken (red dot line; y = 0.1543x - 3.0259 R^2^ = 0.9539).

As shown in Fig 5, the notochord of BTD embryos also shows a slower development than that of TCC embryos as determined by the slope (R^2^ = 0.90 vs. 0.95) of the regression lines and the relative lengths at each parallel time points. The length of notochord ranged from 0.5 to 3.49 mm in TCC embryos and from 0.57 to 1.86 mm in BTD embryos during the first 42 h of incubation.

### Early developmental stages in BTD and TCC embryos

**Fig 6:**
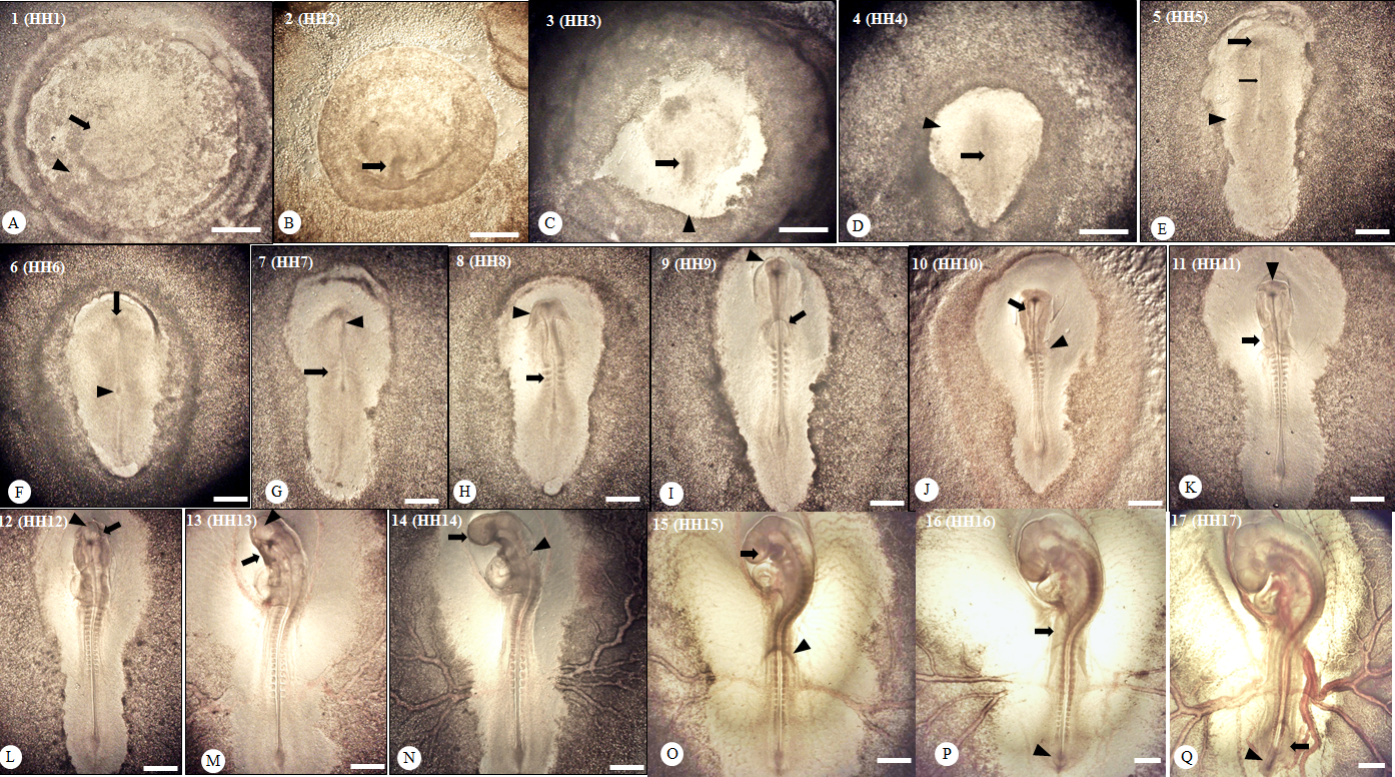
Anatomical structures of developing Brown Tsaiya Duck embryos during the first 72 h of incubation. (A) The area opaca (arrowhead) and pellucida (arrow) are clearly distinguishable at 7 h post-incubation; no other prominent embryonic structures can be observed at this stage. (B) The primitive streak (arrow) starts budding out and becomes visible at 14 h post-incubation; (C) an intermediate or growing streak (arrow) stems from the marginal zone (arrowhead), extending out at 19 h post-incubation; (D) the streak becomes longer (arrow) and the area pellucida forms a V-shape or an ice-cream cone shape (arrowhead) at 28 h post-incubation; (E) the head process emerges (arrow) at 34 h post-incubation while the notochord extended posteriorly from the head position (thin arrow) to the Hensen’s node, and the area pellucida becomes elongated along the anterio-posterial axis (arrowhead); (F) the headfold (arrow) becomes visible and Hensen’s node repositions around the center of the embryo (arrowhead) by 31 h post-incubation; (G) the first 3 pairs of somites (arrow) and the neural fold (arrowhead) appear by 33 h post-incubation; with the lengthening of the notochord, the Hensen’s node starts to migrate caudally accompanied with the shortening of the primitive streak; (H) around 4–5 somite pairs (arrow) and the subcephalic pocket of the embryo are visible (arrowhead) at 36 h post-incubation; (I) the optical vesicles (arrowhead) and the paired heart primordia (thin arrow) are formed at 42 h post-incubation; (J) the forebrain region of three primary brains (arrow) and the omphalomesenteric veins (arrowhead) become distinguishable by 46 h post-incubation; (K) the heart loop is bent to the left (arrow) and the neuropore is not yet closed (arrowhead) 52 h post-incubation; (L) the optical vesicles (arrow) and proencephalon (arrowhead) are forming by 58 h post-incubation; (M) the head is turning to the left (arrow) and the telencephalon (arrowhead) enlarge by 60 h post-incubation; (N) the head completely turns to the left (arrow) and the auditory pit (arrowhead) is prominent 63 h post-incubation; (O) By 65 h, the optic cup was completely formed (arrow) and the amnion extended to somites 14–15 (arrowhead), while the crown-tail axis further curling up; (P) with increasing body size, the primordia wing buds first appeared as a tiny scratch (arrow) and tail bud formed a short cone-shaped structure (arrowhead) by 70 h; (Q) The body size further increased and peripheral vessels gradually filling in with blood cells (fine arrows); the leg buds are lifted off and appeared as fine crest (arrow) and tail bud appeared as a small bulge and slightly bent ventrally (arrowhead) by the end of 72 h. Numerical numbers 1–17 and HH1-HH17 represent different developmental stages in BTD and HH staging system, respectively. Bright field, scale bar = 0.5 mm.

The established developmental stages of BTD are shown in Table 3. Two distinct anatomical zones, the area opaca and the area pellucida, for primitive streak formation were observed during the first 12 h of incubation before the occurrence of the streak. After reaching its maximal length, the streak length of BTD embryos was then gradually shortening when the headfold was form by 31–36 h post-incubation. Cardiogenesis was initiated by the appearance of a paired primordial heart at 36 h post-incubation. The five neuromere segments emerged during 46–52 h of incubation, and the optic cups were completely formed around 62–65 h post-incubation. During 32–72 h post-incubation, the progression of somite number was observed reaching 16–17 somite pairs, i.e., 32–34 somites, which is comparable to earlier embryonic stages HH7-HH17 in chicken. Representative anatomical structures at early developmental stages of BTD embryos are also shown in Fig 6.

**Table 3:** Staging with key developmental features of Brown Tsaiya Duck embryos during the first 72 h of incubation.

Stage	Hour (h) post-incubation	Stage description (relative to HH staging system)
1 (HH1)	1–12	The area pellucida was easily distinguished from the area opaca; small clusters of cells emerged and formed a reticular structure; hypoblasts formed; the marginal zone, the thick area at posterior zone, became prominant between the two-layered germinal discs.
2 (HH2)	10–14	Initial streak formation: the primitive streak initiated as a short conical condensation in the area pellucida (0.34–0.61 mm in length); margins of the marginal zone and the primitive streak were not clearly separated yet.
3 (HH3)	13–24	Elongation of the intermediate or growing streak: the primitive streak extended to the center of area pellucida and became a dense bulge 19 h post-incubation. The primitive streak was separated from the margin of the marginal zone and became a rostral-caudal mid-line within the margin of the blastoderm, where the primitive groove was still not clearly visible (0.39–1.07 mm in length).
4 (HH4)	25–32	Formation of the definite or full-length streak: the primitive streak reached its maximal length (0.73–1.25 mm, average length = 0.93 mm). The primitive pit, primitive groove, and Hensen’s node were prominent with no head process visible yet. The primitive streak ultimately extended about 2 to 3 times of its initial length. The area pellucida became somewhat V-shaped by 28 h post-incubation.
5 (HH5)	25–35	The head process became visible but no headfolds formed yet by 34 h post-incubation. The notochord extended from the head position caudally to Hensen’s node. The area pellucida became elongated along the anterio-posterial axis. The initial neurulation; the neural plate became clearly visible by the end of this period.
6 (HH6)	31–36	Headfolds appeared by 31 h of incubation, but no somite formation was identified yet. Hensen’s node shifted to the central area of the embryo between the notochord and the primitive streak.
7 (HH7)	32–34	Initiation of somitogenesis; one to 3 pairs of somites were formed by 33 h of incubation and neural folds appeared on both sides of the neural plate in the cephalic region. The notochord prolonged and the Hensen’s node started to migrate caudally accompanied with the shortening of the primitive streak.
8 (HH8)	32–36	The 4^th^ to 5^th^ pair somite stage; the neural folds curved dorsally and met around the midbrain. The subcephalic pocket became visible 36 h post-incubation.
9 (HH9)	36–42	The 6^th^ to 9^th^ pair somite stage; the primary optical vesicles were first recognized by 42 h of incubation, and paired heart primordia started to fuse. The neural groove closed and formed the neural tube accompanied by the increasing size of the subcephalic pocket 41 h after incubation.
10 (HH10)	43–46	The 10^th^ to 11^th^ pair somite stage; the heart loop slightly bent to the left and the three primary brain vesicles were first visible by 43 h after incubation. The omphalomesenteric veins began to grow from the caudal end of the heart by 46 h of incubation.
11 (HH11)	46–52	The 12^th^ to 14^th^ pair somite stage; five neuromeres of the hindbrain were visible and the neuropore was not closed yet. The optic vesicles constricted at the base by 48 h of incubation and the heart was fully bent toward the left by 52 h of incubation.
12 (HH12)	52–58	The 16^th^ to 17^th^ pair somite stage; the neuropore was closed and the enlargement of proencephalon (the anterior part of the head) occurred; the primary optic vesicles and optic stalks were formed. The auditory pit was wide open by 57 h; the heart developed into slightly S-shaped by 58 h, and the head fold partially covered the forebrain.
13 (HH13)	54–60	The 18th to 19th pair somite stage; the head was turning to the left and the telencephalon was distinctly enlarged; the head fold from the amnion gradually covered the forebrain, midbrain and the anterior part of hindbrain.
14 (HH14)	59–63	The 21^th^ to 23^th^ pair somite stage; cranial and trunk flexures of embryos developed; By 63 h post incubation, opening of the auditory pit was defined; the amnion was extending close to somites 7 to 10.
15 (HH15)	62–65	The 24^th^ to 25^th^ pair somite stage; the head flexure was progressively evident; the optic cup was completely formed and the amnion extended from somites 7 to 15.
16 (HH16)	64–72	The 26^th^ to 32^th^ pair somite stage; the amnion grew and extended to somites 10 to 18; the primordia wing bud formed as a fine crest, whereas the leg bud was still invisible; tail bud appeared as a short straight cone; epiphysis was not formed yet.
17 (HH17)	71–72	The 32^th^ to 34^th^ pair somite stage; the head and body flexures were pronounced; the leg buds is lifted off and appeared as fine crest; tail bud appeared as a small bulge and bent somewhat ventrally. The amnion appeared but the allantois was not yet formed.

Table 4 shows the staging of TCC and BTD embryos during the first 72 h of incubation, which are presented in parallel to the HH staging system in hourly basis post-incubation. Compared to the HH staging system, Stages 14–16 of BTD embryos were largely equivalent to the Stage 19 of TCC embryos.

**Table 4:** Staging Taiwan Country Chicken (TCC) and Brown Tsaiya Duck (BTD) embryos by the incubation time (h) relative to Hamburger and Hamilton (HH) and Dupuy (D-) staging system during the first 72 h of incubation.

		Hour (h) post-incubation		
The HH stage	D-staging	HH	TCC	BTD
1	13–14	0–5	0–5	0–12
2	15	6–7	6–7	10–14
3	16	12–13	12–13	13–24
4	17	18–19	18–19	25–29
5	18	19–22	19–22	25–31
6	19	23–25	22–25	31–35
7	20	23–26	24–26	33–34
8	21	26–29	26–29	32–36
9	22	29–33	29–33	36–42
10	23	33–38	33–39	43–46
11	24	40–45	40–45	45–48
12	25	45–49	45–49	50–52
13	26	48–52	48–52	54–56
14	27	50–53	50–53	60–64
15	28	50–55	50–55	64–68
16	29	51–56	52–56	69–72
17	30	52–64	52–64	71–72
18	31	65–69	65–69	N/A
19	32	68–72	68–72	N/A

N/A: data not available.

## Discussion

Brown Tsaiya Duck *(Anas platyrhynchos)* is a native breed of major egg layer ducks in Taiwan, and the female BTD is bred with the male Muscovy duck to produce the Mule duck for meat production [18–19]. Taiwan Country Chicken is also one of the native avian breeds in Taiwan, bred and preserved in Genetics and Breeding Resource Center of Country Chicken, National Chung Hsing University, as one of the valuable resources among native poultry species. The TCC used in this study were bred by using the NCHU B line with the NCHU S line; both lines are important meat type chickens that share more than 60% local poultry market in Taiwan.

In theory, the egg weight might affect the measurements in some growth parameters such as hatchability or chick hatch-weight of the developing chick embryo [20–22], but their final hatching day, being around 21 days post-incubation, remains the same within avian species [6, 12, 23]. However, to avoid individual variations, only eggs of a similar size were selected for the present study.

In previous studies, Liu *et al*. [24] have shown that the fertilization rate of BTDs ranges from 79.1% to 86.1% and Rouvier *et al*. [25] have shown that of Kaiya ducks (Pekin × White Tsaiya) being 63%-83%. In the present study the fertilization rate of BTDs (85.7%) was also within the normal range as abovementioned; even with a slightly lower incubation temperature (37.2°C) the hatching time (28 d) of BTDs was similar to that of Pekin ducks and Muscovy ducks which ranged from 28 to 30 d after the onset of incubation. In TCC embryos, the average fertilization rate was also close to that of the broiler chicken (TCC: 90.3% *vs*. broiler: 90.9–92.4%) [25]. In contrast, it appears that the fertilization rates of TCC were slightly lower than those of Leghorn layers (94.7%) [26] but higher than those of guinea fowls (33.9 g) [15]. It is most likely due to the differences between the types of chicken breeds [18].

During the first 72 h of development, the major structural milestone of gastrulation in avian embryos is the formation of the primitive streak, which defines the anterior-posterior axis of the embryo and gives rises to the majority of endodermal and mesodermal tissues during later development [27]. It first appears as a regional thickening of epiblast cells posterior to the embryo and then elongates toward the future head region, extending 60–75% longitudinal length of the area pellucida. After reaching its full length, the streak starts to regress by shifting from the area pellucida to a more posterior position while the head process becomes visible. In the present study, we found that the formation of the primitive streak in TCCs differed in their timing compared to BTDs. The earliest difference between TCC and BTD embryo development laid prior to the HH2 stage, *i*.*e*., the formation of the Koller’s sickle and the hypoblast. The Koller’s sickle is the embryonic structure inducing the primitive streak and Hensen's node [28– 29], and was not observed in the duck embryo by 0–12 h post-incubation; it is normally observed in the chicken embryo at 0–5 h post-incubation. Dupuy *et al*. [10] reported that the sign of hypoblast formation is not as evident in duck embryos as in both chicken and turkey embryos. In most duck embryos, polyingressing cells derived from the epiblast that incorporate into the hypoblast are evenly distributed over the ventral area pellucida. At this stage the duck embryo was nearly symmetrical prior to achieving a more polarized appearance during later development. Therefore, formation of the primitive streak was apparently delayed (4–6 h) in BTD embryos compared to that in TCC embryos. In contrast, the profile and timing of the primitive streak development in BTD embryos are consistent across duck species reported. For example, the growing or intermediate primitive streak extended to the center of area pellucida after 19–24 h post-incubation which was similar to that in Penkin ducks observed by Koltofen [9] and Dupuy *et al*. [10]. However, chicken embryos only spent 12–13 h post-incubation to reach the same stage of development in TCC and HH embryos.

Due to its relatively slower development of the primitive streak compared to TCC embryos, the early organogenesis, including development of somites, the nervous system, the cardiovascular system, and limb buds of BTD embryos would conceivably require a longer time to reach the same developmental stages as in TCC embryos.

The allantois, an extraembryonic membrane and a precursor of the primitive vasculature derived from the mesoderm [30], began to take shape on day 3 of incubation in TCC embryos, but it was still not found in BTD embryos. It is known when the allantois vesicle enlarges, the mesodermal layer of the allantois fuses with the adjacent mesodermal layer of the chorion to form the chorioallantoic membranes (CAM), which can rapidly expand and generate a rich vascular network to form an efficient interface for gas and waste exchanges [31]. Any undesired endogenous and exogenous factors would conceivably alter or retard the development of the CAM. Therefore, this unique extraembryonic structure has been used for screening drug toxicity by monitoring its hemorrhage state or dynamic wax-and-wane of angiogenesis due to its drug sensitivity [32]. Similarly, the CAM of duck embryos could also be used for the same purpose; however, only BTD embryos beyond day 3 of incubation would be most suitable when the CAM becomes fully vascularized.

The ECTL has been considered to be an immediate indicator for monitoring embryonic growth, and the number of somites has also been a major parameter for staging embryos. In the present study, two regression lines, were established to predict the size of embryos during the first 72 h of development. Apparently, the significantly high R-square values (0.95 and 0.93 for BTDs and TCCs, respectively) of these regression models could provide accurate predictions for the length of ECTL and somite numbers in both TCCs and BTDs. Based on these linear regression lines, the average ECTL per hour in TCC or BTD embryos appeared steadily increased (Fig 3), and the predicted lengths were 7.82 mm in TCC embryos which was longer than that of TCC embryos (*cf*. 5.49 mm) at 72 h post-incubation. In fact, based on our observation the ECTL of TCC and BTD embryos were 8.93 mm and 6.23 mm, respectively. During the first 72 h of development, the length of the BTD embryos (6.23 mm) were found close to that of other ducks, i.e., Khaki Campbell and White Indian Runner (6.2 mm) [11] but smaller than that of Penkin ducks (8–9.5 mm) [9]. The sizes of TCC and BTD embryos can be compared side by side using the ECTL of both species at the same age during the early stage of embryogenesis.

For somitogenesis, during the first 72 h post-incubation, the progression of somitogenesis of the BTD described in the present study was found to be similar to that of the Pekin duck observed by Dupuy [9] and Khaki Campbell and White Indian Runners observed by Koecke [11]; the appearance of the first somite in BTD was by 32–34 h *vs*. 33–36 h in Pekin ducks by and 32–36 h in Khaki Campbell and White Indian Runner ducks. By the same period of developmental stage, the numbers of somite pairs in BTD (32–34) were not much different from other duck species mentioned (29–31). In contrast, the somite numbers of BTD embryos differed from that of the TCC embryos. The delayed somitogenesis in BTD embryos was likely due to the slowed development in the vascularization of the CAM as mentioned above. The previous study has indicated that a pair of somites are formed every 90 min, and the completion of 52 somite pairs can last for 5 days during the early development of chicken embryos [33]. However, the above-mentioned events have not been well described in duck embryos particularly in BTDs. A longer incubation time may be necessary to investigate into the precise developmental time course and the cyclic clock of somitogenesis of duck embryos as in chicken embryos [34].

The trends of developing primitive streaks in TCC and BTD embryos that were presented with quadratic regression lines (Fig 4). In the beginning of primitive streak formation, TCC embryos exhibited a faster development than that in BTD embryos. The primitive streak in TCC embryos disappeared after 42 h of incubation, but it remained in BTD embryos. Based on the R^2^ values of the established regression lines, it appeared that the quadratic regression model was of a better prediction on the primitive streak length in BTD embryos than in TCCs (*cf*. 0.87 *vs*. 0.73). When the length of primitive streaks was predicted using the quadratic equations, maximal lengths of the primitive streak occurred at 18.94 h and 26.98 h in TCC and BTD embryos, respectively. According to our observation, maximum length of the primitive streak appeared by 18–19 h and 25–27 h in TCC and BTD embryos (Table 2), respectively.

Furthermore, we found that patterns of notochord development in TCC and BTD embryos could be predicted by two linear regression lines as well (Fig 5). When extrapolated with the established regression equations, the notochord length of BTD embryos approximately took 14.9 h more incubation time to be equivalent to that of the TCC embryo. For instance, to reach 3 mm of the notochord length, 39.05 and 53.95 h of incubation were required for TCC and BTD embryos, respectively.

Our observation showed that the regression of the primitive streak occurred after 22 h and 28 h post-incubator in TCC and BTD embryos, respectively; in the meanwhile, the notochord became clearly visible and gradually elongating. Therefore, the growth of the notochord parallels to the regression of the primitive streak as previously reported [35].

During the first 72 h of incubation, most of the major organ rudiments had formed in avian embryos. Among the chicken embryo no differences could be observed between TCC and other chicken embryos reported by Sellier (2006) [12], as well as of the HH staging system (HH 20). However, the TCC embryos developed slightly faster than those of Guinea fowl embryos (HH 15). Although the BTD embryos developed approximately 15–17 h slower than those of TCC embryos. In the present study, the early stages of BTD embryos were similar to that of Pekin duck embryos (HH17) but slightly faster than those of Muscovy ducks and Mulard ducks (HH17 vs. HH12 and HH14; approximately 18 h) [10, 12]. Unlike the TCC embryos, BTD embryos required 33–36 h to enter the HH7 stage (*cf*. 24–26 h in TCC embryos) and could only reach Stages HH14-HH16 after 72 h of incubation (*cf*. HH19 in TCC embryos).

Although the pace of development was generally slower in BTD embryos, the basic phenomena and the nature of the overall developmental phases remained similar to that of the chicken embryos. To characterize the distinct changes, the course of development was also mandatorily classified into 17 stages for BTD embryos (Table 3). More specifically, the BTD embryos developed 10 h (2%) slower than those of TCC embryos by the first 24 h of incubation. After 72 h of incubation, BTD embryos showed approximately 15–17 h (3.3%) slower than those of TCC embryos. It is known that BTD embryos take 7 days (33%) more to hatch than chicken embryos by the end of embryogenesis. Given that a slightly delay at each developmental stage can later add up to approximately one week delay over the whole incubation period, it is still unclear whether each developmental event of BTD embryos is proportionally setback throughout the entire hatching development, or some of the unique developmental features specifically prolong the overall development in BTD embryos during the first 72 h of incubation.

Embryonic stages of TCC and BTD embryos were compared based on the HH staging system (Table 4). We found that BTD embryos, on average, was approximately 8 h slower than TCC and HH embryos during Stages 1 to 10, but after Stages 11 to 13 (by neurulation), the developmental time course of BTD became faster and approximately only 3–5 h slower than that in TCC and HH embryos. Nevertheless, up to Stage 14 TCC and HH embryos speeded up the rate of development and became approximately 15–17 h faster than BTD embryos.

For later development, the appearance of beaks and webbed feet of embryos are apparently distinct between chicken and ducks (development beyond 72 h). These are also major parameters for staging and those contribute to the differential time course of development till hatching between chickens and waterfowls. For future work, it is of necessity to investigate the unique features of developing BTD embryos by a longer (> 72 h) incubation time to characterize later embryogenesis and development in BTDs.

In conclusion, in spite that TCC and BTD embryos have shared some similarities with the embryos staged by the HH system, the present study describes, for the first time, more precise timings of the emerging embryonic features with a side-by-side comparison between the two species during the first 72 h of embryogenesis. To our best knowledge, it is also the first report that implements the established regression lines to depict early embryogenesis in Brown Tsaiya ducks with several well-defined embryonic features, such as gastrulation, neurulation, somitogenesis and organogenesis, along the developmental stages of this species.

## Supporting information

S1 Table Summary of key developmental features of Taiwan Country Chicken embryos during the first 72 h of incubation. (PDF)

S1 Fig Anatomical structures of developing Taiwan Country chicken embryos during the first 72 h of incubation. (A) The area pellucida (arrow) and the area opaca (arrowhead) are distinct by 4 h post-incubation. (B) The primitive streak (arrow) first appear by 7 h post-incubation. (C) The intermediate or growing streak (arrow) is visible around 12 h post-incubation. (D) The definitive or full length streak (arrow) is visible by 19 h post-incubation, with a clear primitive groove (thin arrow) and Hensen’s node centering the embryo (arrowhead). (E) The head process (arrow) is taking shape by 22 h post-incubation; Hensen’s node and definitive primitive streak are clearly visible. (F) The headfold (arrow) becomes visible 25 h post-incubation; (G) the first pair of somites (arrow) appear around 25 h post-incubation. (H) Four somites (arrow) and neural fold (arrowhead) appear by 26 h post-incubation; (I) the optical vesicles (arrow) and the paired primordia of the heart (arrowhead) formed 30 h post-incubation. (J) The heart loops slightly bend to the left (arrow) and the three primary brains (arrowhead) are visible 33 h post-incubation; (K) the five neuromeres (arrow) are visible and optic vesicles (arrowhead) are constricted around 42 h post-incubation; (L) the heart (arrow) is forming into a slightly S-shaped and the neuropore (arrowhead) is closed by 45 h post-incubation. (M) The head (arrow) bends to the left and the telencephalon (arrowhead) is enlarged around 48 h post-incubation; (N) the head completely bends to the left (arrow) and the margin of amnion (arrowhead) is visible by 51 h post-incubation. (O) The optic cup (arrow) is completely formed 54 h post-incubation. (P) The wing (arrow) and leg buds (arrowhead) develop by 52 h post-incubation. (Q) The auditory pit (arrow) and the amnion (arrowhead) are present by 55 h post-incubation. (R) The leg (arrow) and tail buds (arrowhead) are more prominent by 65 h post-incubation. (S) The enlargement of the leg (arrow) and wing buds (arrowhead) becomes prominent by 72 h post-incubation. Numerical numbers 1–19 and HH1-HH19 represent different developmental stages in TCC and HH staging system, respectively. Bright field, scale bar = 0.5 mm.
(TIF)
