# Supplementary material for: Characterizing early embryonic development of Brown Tsaiya Ducks (Anas platyrhynchos) in comparison with Taiwan Country Chicken (Gallus gallus domestics)
Source: PLoS One. 2018 May 9;13(5):e0196973. doi: 10.1371/journal.pone.0196973 (PMC5942818; doi:10.1371/journal.pone.0196973)
Supplement: S1 Table — (PDF) [file pone.0196973.s001.pdf]

S1 Table. Summary of key developmental features of Taiwan Country Chicken embryos during the first 72 h of incubation.

Stage	Hours post-incubation	Stage description relative to the HH staging system (Taiwan Country Chicken, NCHU B line × S line)
1 (HH1)	0-5	The prestreak stage; two-layered germinal disc was formed.
2 (HH2)	6-7	The initial primitive streak, the beginning of streak formation: the primitive streak occurred as a short conical condensation in the area pellucida.
3 (HH3)	12-13	Intermediate streak elongation: the primitive streak extended to the center of the area pellucida; no primitive groove appeared yet.
4 (HH4)	18-19	Definitive streak formation: the primitive streak reached its maximal length; Hensen’s node, primitive pit, and primitive groove were present; no head process was observed yet.
5 (HH5)	19-22	Visible head process; no head fold was observed yet. Hensen’s node and definitive primitive streak are clearly visible
6 (HH6)	22-25	Appearance of the head fold; no somite was observed yet.
7 (HH7)	24-26	The first somite appeared; neural folds and the neural plate were observed.
8 (HH8)	26-29	The 4-somite stage; neural folds from both sides united around the midbrain.
9 (HH9)	29-33	The 7-somite stage; visible primary optical vesicles; the progressive of fusion of the paired heart primordia were began into tubes unite.

S1 Table. (Continued)

10 (HH10)	33-39	The 10-somite stage; cranial flexure gradually appeared; 3 distinguishable primary brain vesicles (a mesencephalon; midbrain, behind the pros encephalon; forebrain and a rhomb encephalon; hindbrain); The heart slightly bent to the right.
11 (HH11)	40-45	The 13-somite stage; cranial flexure was prominent; 5 neuromeres formed; optic vesicles constricted at the base; heart loop completely bent to the right.
12 (HH12)	45-49	The 16-somite stage; head turning to the left; anterior neuropore closed; telencephalon initiated; primary optic vesicles and optic stalk established; visible auditory pit wide open; heart formed a slightly S-shaped; head fold of the amnion covered the forebrain.
13 (HH13)	48-52	The 19-somite stage; distinct enlargement of the telencephalon; no hypophysis being observable yet; head folds of amnion are cover up the forebrain until anterior part of the hindbrain.
14 (HH14)	50-53	The 22-somite stage; cranial and trunk flexures of embryo developed; visceral arches 1 and 2 and visceral clefts 1 and 2 were visible; the primary optic vesicle and lens placode were formed; opening of the auditory pit constricted; the amnion enlarged and extended to somites 7 – 10.
15 (HH15)	50-55	The 24 to 27-somites stage; the 3rd visceral arch and cleft became visible; the optic cup was completely formed.
16 (HH16)	52-56	The 26 to 28-somite stage; the primordia of wings and legs were present; epiphysis was not formed yet.
17 (HH17)	52-64	The 29 to 32-somite stage; lateral body folds extended around the entire circumference of embryos; cranial flexure unchanged but the cervical and trunk flexures became more prominent than the previous stages; the tail-bud bent ventrally; the amnion may be variably observable; the allantois had not formed yet.

S1 Table. (Continued)

18 (HH18)	65-69	The 30 to 36-somite stage; leg buds were larger than wing buds; trunk flexure shifted to lumbal region; tail bud turned to the right; the amnion closed and the allantois remained a small, thick-walled bud; the 4th visceral arch was undefined and the maxillary process was still absent
19 (HH19)	68-72	The 37 to 40-somite stage; the limb buds were symmetrical; trunk flexure disappeared; the tail-bud curved and pointed forward; visceral arches appeared: the maxillary process appeared, enlarged and became distinctive; the 1st visceral cleft open as a narrow slit, but the 4th visceral cleft did not open; the eyes remained unpigmented; the allantois was of variable sizes and without vascularization.
