# Supplementary figures and images for: Characterizing early embryonic development of Brown Tsaiya Ducks (Anas platyrhynchos) in comparison with Taiwan Country Chicken (Gallus gallus domestics)
Source: PLoS One. 2018 May 9;13(5):e0196973. doi: 10.1371/journal.pone.0196973 (PMC5942818; doi:10.1371/journal.pone.0196973)

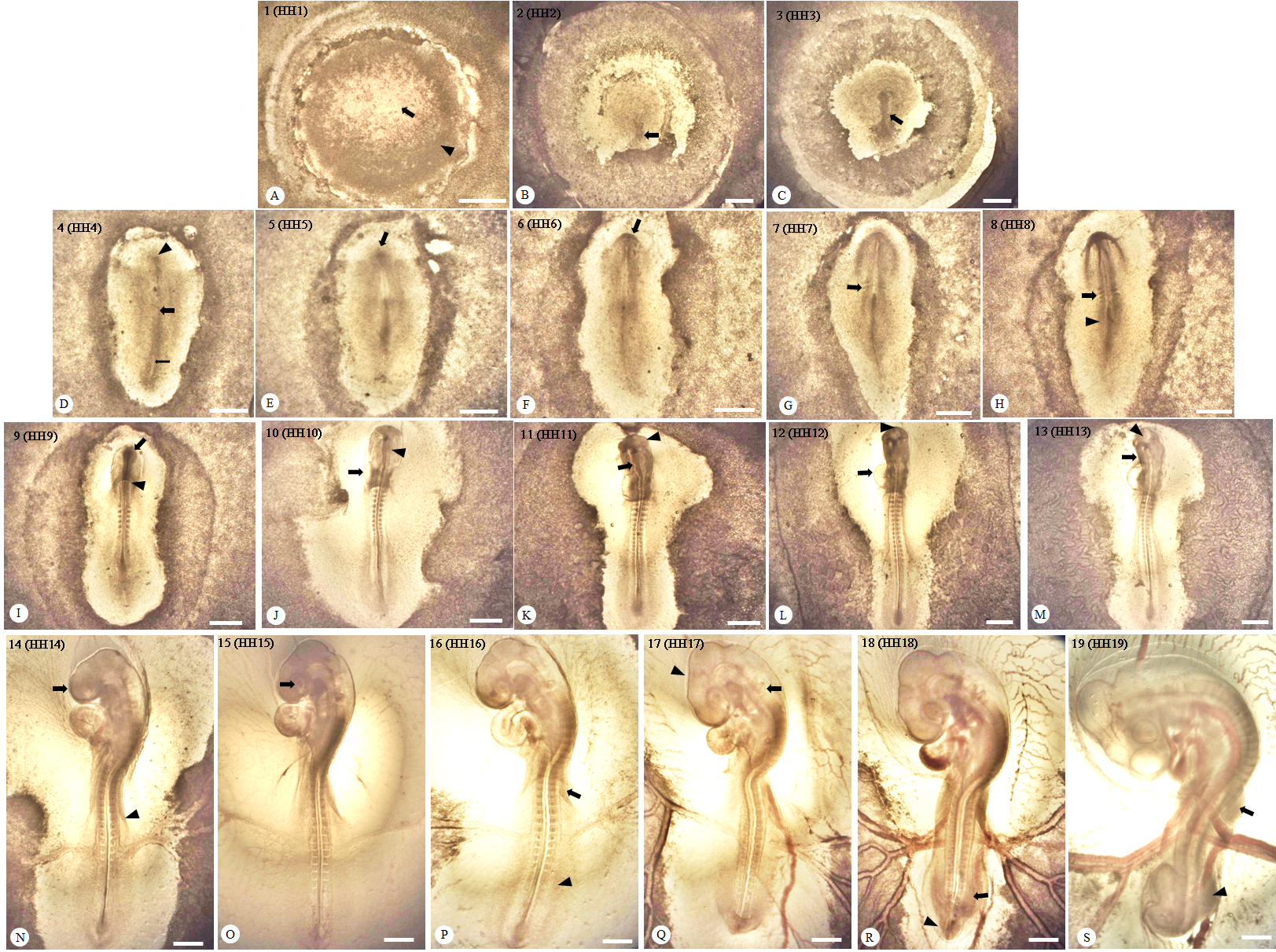

Supplement: S1 Fig — (A) The area pellucida (arrow) and the area opaca (arrowhead) are distinct by 4 h post-incubation. (B) The primitive streak (arrow) first appear by 7 h post-incubation. (C) The intermediate or growing streak (arrow) is visible around 12 h post-incubation. (D) The definitive or full length streak (arrow) is visible by 19 h post-incubation, with a clear primitive groove (thin arrow) and Hensen’s node centering the embryo (arrowhead). (E) The head process (arrow) is taking shape by 22 h post-incubation; Hensen’s node and definitive primitive streak are clearly visible. (F) The headfold (arrow) becomes visible 25 h post-incubation; (G) the first pair of somites (arrow) appear around 25 h post-incubation. (H) Four somites (arrow) and neural fold (arrowhead) appear by 26 h post-incubation; (I) the optical vesicles (arrow) and the paired primordia of the heart (arrowhead) formed 30 h post-incubation. (J) The heart loops slightly bend to the left (arrow) and the three primary brains (arrowhead) are visible 33 h post-incubation; (K) the five neuromeres (arrow) are visible and optic vesicles (arrowhead) are constricted around 42 h post-incubation; (L) the heart (arrow) is forming into a slightly S-shaped and the neuropore (arrowhead) is closed by 45 h post-incubation. (M) The head (arrow) bends to the left and the telencephalon (arrowhead) is enlarged around 48 h post-incubation; (N) the head completely bends to the left (arrow) and the margin of amnion (arrowhead) is visible by 51 h post-incubation. (O) The optic cup (arrow) is completely formed 54 h post-incubation. (P) The wing (arrow) and leg buds (arrowhead) develop by 52 h post-incubation. (Q) The auditory pit (arrow) and the amnion (arrowhead) are present by 55 h post-incubation. (R) The leg (arrow) and tail buds (arrowhead) are more prominent by 65 h post-incubation. (S) The enlargement of the leg (arrow) and wing buds (arrowhead) becomes prominent by 72 h post-incubation. Numerical numbers 1–19 and HH1-HH19 represent [file pone.0196973.s002.tif]
